# Fault Diagnosis from Raw Sensor Data Using Deep Neural Networks Considering Temporal Coherence

**DOI:** 10.3390/s17030549

**Published:** 2017-03-09

**Authors:** Ran Zhang, Zhen Peng, Lifeng Wu, Beibei Yao, Yong Guan

**Affiliations:** 1 College of Information Engineering, Capital Normal University, Beijing 100048, China; zhangran@cnu.edu.cn (R.Z.); yaobeibei@cnu.edu.cn (B.Y.); guanyong@cnu.edu.cn (Y.G.); 2 Beijing Engineering Research Center of High Reliable Embedded System, Capital Normal University, Beijing 100048, China; 3 Beijing Advanced Innovation Center for Imaging Technology, Capital Normal University, Beijing 100048, China; 4 Information Management Department, Beijing Institute of Petrochemical Technology, Beijing 102617, China; zhenpeng@bipt.edu.cn

**Keywords:** faults diagnosis, deep neural networks, raw sensor data, temporal coherence

## Abstract

Intelligent condition monitoring and fault diagnosis by analyzing the sensor data can assure the safety of machinery. Conventional fault diagnosis and classification methods usually implement pretreatments to decrease noise and extract some time domain or frequency domain features from raw time series sensor data. Then, some classifiers are utilized to make diagnosis. However, these conventional fault diagnosis approaches suffer from the expertise of feature selection and they do not consider the temporal coherence of time series data. This paper proposes a fault diagnosis model based on Deep Neural Networks (DNN). The model can directly recognize raw time series sensor data without feature selection and signal processing. It also takes advantage of the temporal coherence of the data. Firstly, raw time series training data collected by sensors are used to train the DNN until the cost function of DNN gets the minimal value; Secondly, test data are used to test the classification accuracy of the DNN on local time series data. Finally, fault diagnosis considering temporal coherence with former time series data is implemented. Experimental results show that the classification accuracy of bearing faults can get 100%. The proposed fault diagnosis approach is effective in recognizing the type of bearing faults.

## 1. Introduction

Monitoring machinery health conditions is crucial to its normal operation. Recognizing the deficiencies of machinery contributes to the control of the overall situation. Fault diagnosis models based on data-driven methods are a great advantage since that they require no physical expertise and provide accurate and quick diagnosis from data which are easily obtained by sensors. Traditional data-driven fault diagnosis models are usually based on signal processing methods and some classification algorithms. Signal processing methods are mainly used to decrease noise and extract features from the raw data. In this field, time domain feature methods [1,2,3], including Kernel Density Estimation (KDE), Root Mean Square (RMS), Crest factor, Crest-Crest Value and Kurtosis, frequency domain features [4] such as the frequency spectrum generated by Fourier transformation, time-frequency features obtained by Wavelet Packet Transform (WPT) [5] are usually extracted as the gauge of the next process. Other signal processing methods such as Empirical Mode Decomposition (EMD) [6], Intrinsic Mode Function (IMF), Discrete Wavelet Transform (DWT), Hilbert Huang Transform (HHT) [7,8], Wavelet Transform (WT) [9] and Principal Component analysis (PCA) [10] are also implemented for signal processing. These signal processing and feature extraction methods are followed by some classification algorithms including Support Vector Machine (SVM), Artificial Neural Networks (ANN), Wavelet Neural Networks (WNN) [11], dynamic neural networks and fuzzy inference. These classification algorithms are usually used to recognize faults according to the extracted features. Yu et al. [7] used Window Marginal Spectrum Clustering (WMSC) to select features from the marginal spectrum vibration signals by HTT and adopted SVM to classify faults. Wang et al. [8] used the statistical locally linear embedding algorithm to extract low dimensional features from high dimensional features which are extracted by time domain, frequency domain and EMD methods. Regression trees, K-nearest-neighbor classifier and SVM were adopted as the classifiers, respectively. Li et al. [12] used Gaussian-Bernoulli Deep Boltzmann Machine (GDBM) to learn from statistical features of time domain, frequency domain and time-frequency domain. GDBM was also taken as the classifier. Cerrada et al. [13] used a multi-stage feature selection mechanism to select best set of features for the classifier. These researches focus on the steps of feature extraction and selection.

However, these fault diagnosis approaches suffer from some drawbacks. Firstly, applying noise decreasing and feature extraction methods to practical issues properly requires specific signal processing expertise. Specific circumstances call for specific signal processing methods which depend on signal and mathematics expertise; Secondly, the performances of these classifiers completely depend on the features which are extracted from time series signals. Although appropriate features help the decision and recognition, ambiguous features will mislead the model. Thirdly, the feature extraction methods will definitely lose some information such as the temporal coherence of time series data which cannot be ignored.

This paper presents a novel model based on DNN to recognize the raw sensor signals, which are time series data. It has been proved that Deep Learning [14], or Deep Neural Networks are able to reduce the dimensionality and learn characteristics from nonlinear data in the fields of image classification [15], speech recognition [16] and sentiment classification [17]. There are great advantages in the fact that the model can learn directly from the raw time series data and make use of the temporal coherence. The merits of the proposed model are as follows: (1) pretreatments such as noise decreasing and specific time domain and frequency domain feature extraction methods are not needed since the proposed model is competent for the task of learning characteristics from raw time domain signals adaptively; (2) the temporal coherence of time series data is taken into consideration in the proposed model; (3) the proposed model is a supervised learning model and has great abilities of generalization and recognition for the normal and faults data. The classification accuracy on bearing vibration datasets can be 100%.

Complex models are capable of generalizing well from raw data so data pretreatment(s) can be omitted. Models with simple structure do not perform as well as those with deeper and more complex structures, but they are easy to train because they need less parameters. Complex models can get a better understanding of the data due to their multitude of units and layers, however, complex models do not always perform better than simple models. This paper also discusses how the structure of the model affects the performance.

In order to clearly illustrate the model and their performance, the rest of this paper is grouped together as follows: Section 2 describes the details of the proposed fault diagnosis model. Section 3 describes our experiments and results. Section 4 discusses some details of the model structure and makes some comparisons with traditional methods. Section 5 draws the conclusions.

## 2. Methods

This paper presents a fault diagnosis model based on DNN to recognize defects. Inputs of DNN are raw time domain data instead of time domain or frequency domain features. Outputs are the targets, i.e., the categories of data. A deep neural networks structure model is used to learn local characteristics adaptively from the raw time series data. Then temporal coherence is taken into consideration to make a diagnosis.

### 2.1. Structure of Proposed Fault Diagnosis Model

**Figure 1:**
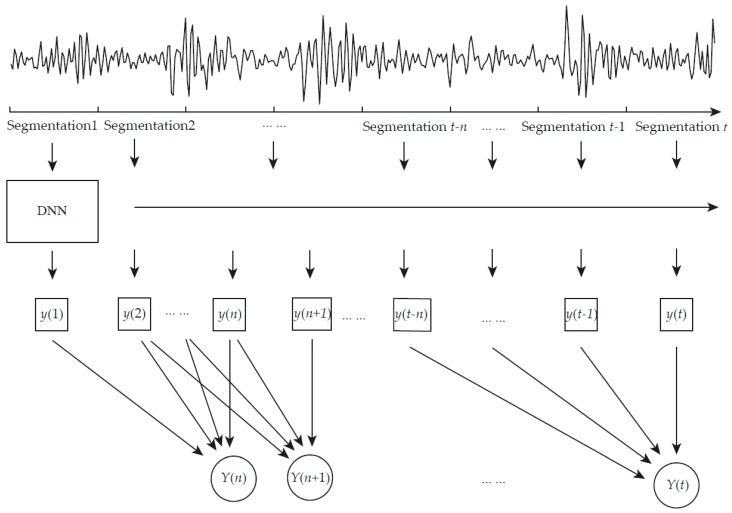
The structure of proposed fault diagnosis model.

As shown in Figure 1, segments of time series data are used as training and testing samples. The size of the segment is set to be a fixed value and it is also the input size of a deep neural networks. The data from time *t1* to *t2* are taken as inputs to the proposed model and the size of the segmentation is *t2 − t1*. In this way, the continuous one dimensional time series data is divided into segments. The time memory is *n*, in other words, *n* is the number of outputs of DNN *y*(*t*) which is temporarily stored for fault diagnosis considering temporal coherence. Therefore, the first output of proposed model which is denoted as *Y*(*n*) is at time *n ×* (*t2 − t1*). Then, for every single length time, which is *t2 − t1*, there is an output *Y*(*t*). For the simplification of representations, *Y*(*t*) denotes the final output of the proposed model at time *t* in rest of this article.

The proposed model is based on the deep structure of neural networks. A logistic function is used as the activation function among input layers and hidden layers. For every segmentation, the model could get a set of output values. Time memory is set to record values of output value *y*(*t*). These sets of former outputs of DNN values are gathered to get final outputs of time considering model via linear transformation:

(1) $$Y(t)=\frac{1}{n}\sum_{T=0}^{n}\lambda_{T}y(t-\;T)$$

In the above equation, *Y*(*t*) is the final outputs at current time and *y*(*t*) is the outputs of DNN at current time. λ is the weights connecting the output units of DNN *y*(*t*) and the final output units of proposed model *Y*(*t*). *n* is the number of DNN outputs which the proposed model takes into account. It can also be considered as the time length.

### 2.2. Deep Neural Networks

Deep learning is firstly introduced in image classification [18] and it is used to reduce the dimensionality of data and recognize images. Deep Learning, or DNN can learn some useful features from the data adaptively without expertise in specific fields. It has received extensive attention in the fields concerned with nonlinear mapping.

DNN is a model of stacked layers of units which are connected layer by layer and there is no connection among the units in the same layer. There are an input layer and an output layer in the model. Also, a few hidden layers are placed between the input layer and output layer. The number of input layer units and output layer units are set according to the dimensionalities of the input data and the target data, respectively. However, there are no strict rules for the settings of the number of every hidden layer’s units. There are nonlinear relationships between the adjacent layers. They are defined as follows:

(2) $$a_{j}^{m}=\sigma (z_{j}^{m})$$

(3) $$z_{j}^{m}=\sum_{i}w_{ij}a_{i}^{m-1}+b_{j}^{m}$$

where $a_{j}^{m}$ is the activation of neural *j* in layer m, $z_{j}^{m}$ is the sum of bias and linear combination of former activations, $b_{j}^{m}$ is the biases vector of neural *j* in layer *m*. $w_{ji}$ is the weight matrix between layer *i* and layer *j*, σ(z) is the activation function and there are a few kinds of this function as follows:

(4) $$\sigma (z)=1/(e^{-z}+1)$$

(5) $$\sigma (z)=\frac{e^{z}-e^{-z}}{e^{z}+e^{-z}}$$

(6) σ(z)=max(0,z)

Equation (3) is a logistic function and this is mostly used in DNNs. Equation (4) is called the tanh function. The range of output values are from −1 to 1. It is different from the logistic function which is easier to implement in the DNNs sometimes. Equation (5) represents rectified linear units (ReLU). Its output is always positive. In this paper, the logistic function are used as the activation function.

Every unit in the next layer is connected to all units in the formal layer. The activation units of input layer are the input data while output units of last layer are targets. *W* and *b* are the parameters of the model and they are randomly initialized. Thus, the outputs *y* can be calculated layer by layer given the input data *x* and the model’s parameters:

(7) $$a^{1}=x$$

(8) $$a_{j}^{m}=\sigma (\sum_{i}w_{ij}a_{i}^{m-1}+b_{j}^{m-1}),j>1$$

(9) $$y=a^{M}$$

where *M* is the number of layers in the network. The inputs of first layer $a^{1}$ are the inputs of DNN. The outputs of last layer $a^{M}$ are defined as the outputs of DNN. The error can be worked out by contrasting the calculated outputs y with targets t:

(10) $$C(t,y)=\frac{1}{k}\sum_{k}\sum_{i}{\left(t_{i}-y_{i}\right)}^{2}$$

where *C* is the cost function, *k* is the number of training samples and *i* represents the dimension of *y* and *t*. The cost function is used to measure the error and the usual form is squared reconstruction error. The aim of training DNN is to minimize the cost function, in other words, to make the error of outputs close or equal to zero. To minimize it, a gradient descent algorithm is utilized and the gradient is calculated as follows:

(11) $$\nabla C=(\frac{\partial C}{\partial \theta_{1}},\frac{\partial C}{\partial \theta_{1}},...,\frac{\partial C}{\partial \theta_{N}})$$

In the equation, *θ* represents parameters including the weights and biases of the layers in DNN model and the total number of parameters is *N*. Partial derivatives of weights and biases of last layer are first calculated according to the error in the last layer. Then the error is back propagated to former layers and all the partial derivatives are worked out. Once the gradient is computed, parameters are updated by the following rules:

(12) $$w=w+\eta \frac{\partial C}{\partial w}$$

(13) $$b=b+\eta \frac{\partial C}{\partial b}$$

In the equation, η is the learning rate. The process of computing gradient and updating parameters is in an epoch. The epochs can either be set to a fixed number or adjusted according to the performance of networks. We train the networks and fine tune the parameters over and over again until the error of the network has declined to the minimum value.

### 2.3. Cross Entropy Cost Function and Partial Derivations

When training neural networks, gradient descent and back propagation algorithms are utilized. Gradients can be calculated by the partial derivative of the cost function for parameters such as weights and biases. Traditional work chooses the squared reconstruction error (Equation (9)) as cost function. However, in the training process of deep neural networks, this will lead to a saturation problem [19]. In other words, all the partial derivatives will be close to zero so the parameter updating will be slow before the networks achieve the best performance. When the cross entropy, also known as Kullback-Leibler (K-L) divergence, is chosen as cost function, the saturation problem will be avoid.

In the proposed model, cross entropy is taken as the cost function to compute the errors. The dimension of inputs, i.e., the number of input neurons is determined by the size of the inputs data and the outputs dimension equals to the number of data types. The cross entropy cost function is defined as follows:

(14) $$C(t,y)=-\frac{1}{k}\sum_{k}\sum_{i}\left[t_{i}lny_{i}+(1-t_{i})ln(1-y_{i})\right]$$

where *t* are targets, *y* are the outputs of the model, *k* is the number of training samples. The main purpose of training is to minimize the cost function. It is obviously that when the outputs *y* are close to the targets *t*, the value of cost will be close to zero. Also the value of the cross entropy cost function will always be positive. These two properties are fundamental for a proper cost function.

A gradient descent is used to minimize the cost function, so the partial derivations are calculated as follows:

(15) $$\frac{\partial C}{\partial w_{ij}^{m}}=a_{i}^{m-1}e_{j}^{m}$$

(16) $$\frac{\partial C}{\partial b_{j}^{m}}=e_{j}^{m}$$

(17) $$e_{i}^{m}=\sum_{j}w_{ij}^{m+1}e_{j}^{m+1}\sigma^{\prime }(z_{i}^{m})$$

(18) $$e_{j}^{M}=\sum_{k}\left(t_{j}-y_{j}\right)$$

where *k* is the number of training samples, layer M is the last hidden layer. $e_{j}^{m}$ is the error of neural *j* in layer m, *t* are the targets. $a_{i}^{m-1}$ is the activation value of neural *i* in layer *m* − 1. $w_{ij}^{m}$ represents the weight from neural *i* in layer *m* − 1 to neural *j* in layer *m*. While the error is back propagated to the former layer, the partial derivations of weights and biases are calculated layer by layer so the parameters can be updated.

### 2.4. Training of DNN and Fault Diagnosis Steps

**Figure 2:**
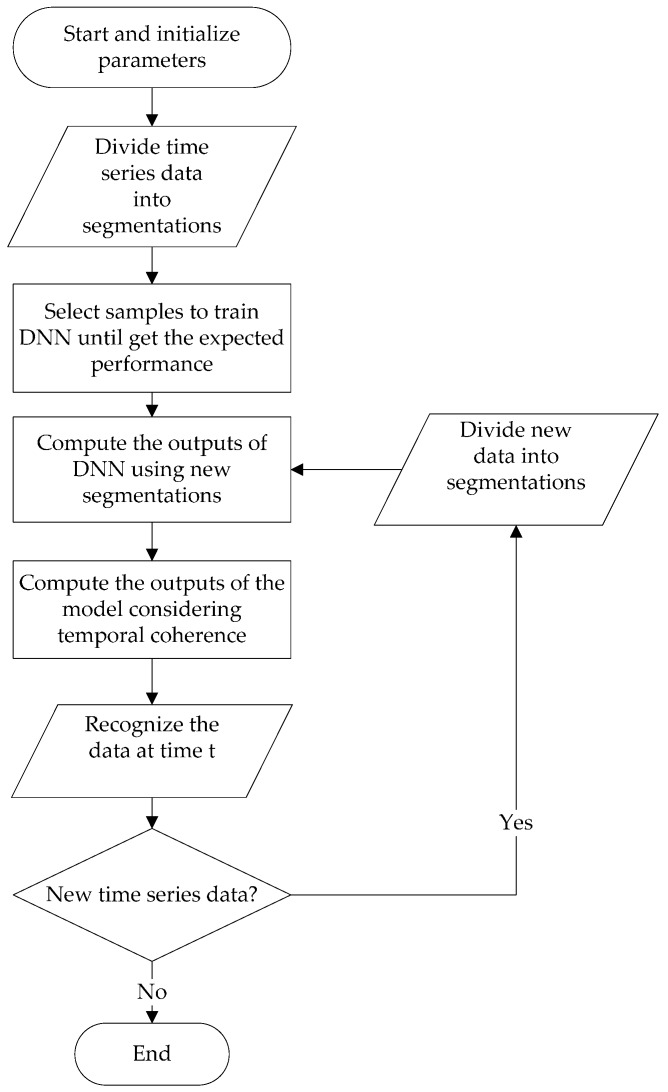
Flowchart of the proposed fault diagnosis approach.

The flow chart of the proposed fault diagnosis approach is shown in Figure 2.

Firstly, the DNN model is trained using historic data and the time memory of the model is set to 0. That is to say, the output of DNN is used as the output of the whole model. In the training process, the time coherence is not utilized so the DNN can independently learn the local characteristics from local vibration data. In this way, the current segmentation will not have any effect of adjacent segments, which is helpful to train a better model. After the training processes come the testing process and fault recognition process. The test data are used to test the classification ability of the trained DNN without considering temporal coherence. In the fault recognition process, the temporal coherence is taken into consideration. The output *Y*(*t*) of proposed model at current time can be computed by the current output of DNN *y*(*t*) and the former outputs *y*(*t − n*) to *y*(*t* − 1) which were stored for a specific time period. The detailed training steps are as follows:

(a) Set the size of segmentation and divide the time series data into segments.
(b) Randomly initialize the parameters of DNN including weights and biases of every hidden and output layer.
(c) Select a group of segments as DNN inputs.
(d) Compute the activations of every layer according to the input raw data, weights, biases.
(e) Cross entropy is used to compute the error of outputs compared to targets.
(f) Use the back propagation algorithm to compute the error of every layer and the gradients of parameters.
(g) Update parameters with the gradients and learning rate.
(h) Repeat steps (c) to (g) until the error of outputs reach the minimum value.

The detailed fault recognition steps are as follows.

(a) Set the model’s memory length n and connection weights λ.
(b) Divide the time series data into segments. If current time is *t* and the size of segmentation is *s*, the segmentation *t* are sample points from *t − s* to *t* and segmentation *t* − *n* are sample points from *t* − (*n* + 1) × *s* to *t* − *n* × s.
(c) Compute the outputs *y*(*t* − *n*) to *y*(*t*) of DNN using segmentation *t* − *n* to segmentation *t* as inputs and store them.
(d) Compute *Y*(*t*) using outputs of DNN from *y*(*t* − *n*) to *y*(*t*) and recognize the data.
(e) When new time series data is available, make them new segments and compute the DNN output. Then, compute the outputs of the proposed model which considers the DNN output history.

## 3. Experiments and Results

### 3.1. Intelligent Maintenance System (IMS) Bearing Dataset

#### 3.1.1. Experimental Apparatus and Data Collection

**Figure 3:**
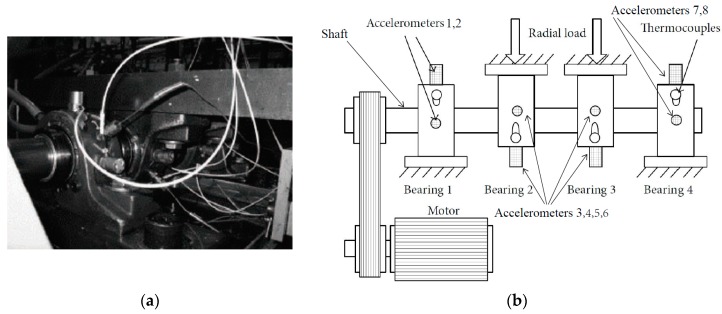
Experimental apparatus. (**a**) is the photo of bearings with sensors. (**b**) is the structure diagram of apparatus.

In order to validate the proposed method, experimental data are applied to test its performance. The dataset is provided by the University of Cincinnati Center for Intelligent Maintenance Systems [20]. The experiment apparatus is shown in Figure 3.

As depicted in Figure 3, there was a shaft on which four bearings were installed. There were eight accelerometers in total, two accelerometers for each bearing. The rotation speed of the shaft was kept constant at 2000 revolutions per minute (RPM). It was driven by an alternating current (AC) motor which was connected to the shaft by friction belts. What’s more, there was a 6000 lbs radial load which was added to the bearings and shaft by a spring mechanism. All four bearings were force lubricated. The bearings were Rexnord ZA-2115 double row bearings. Accelerometers were installed on the bearing housing. The accelerometers were PCB 353B33 High Sensitivity Quartz ICPs. Thermocouple sensors were placed on the bearings as shown in Figure 3. After rotating for more than 100 million revolutions, failures such as inner race defect, outer race failure and roller element defect occurred since the bearings worked for a long period which exceeded the designed life time. Data were collected by a NI DAQ Card 6062E. The sampling rate was set as 20 kHz and every 20,480 data points were recorded in a file. In every 5 or 10 min, the data were recorded and written in a file while the bearings were rotating.

#### 3.1.2. Data Segmentation

**Figure 4:**
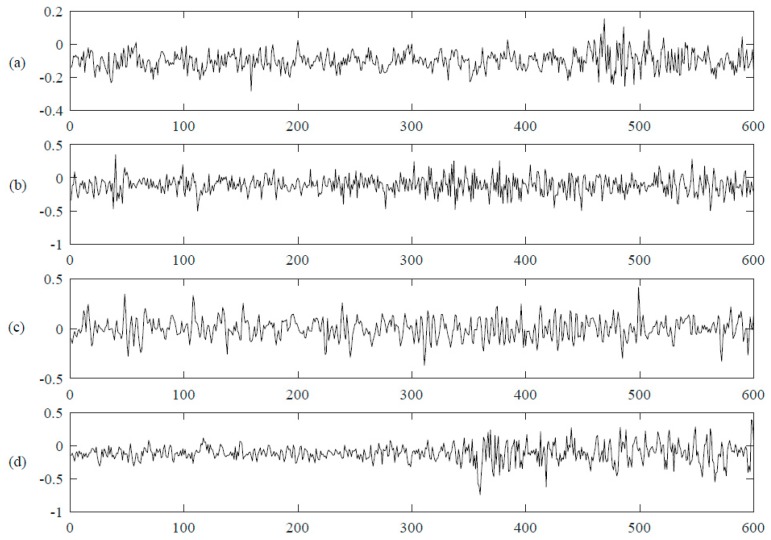
Four kinds of bearing vibration signals. (**a**) is the normal vibration data and the following three lines (**b**–**d**) are three kinds of fault data, inner race fault, outer race fault and roller defect, respectively. The x axis represents time series and y axis represents the collected data value.

Four kinds of data including normal data, inner race defect data, outer race defect data and roller defect data were selected. There are 20,480 data points in each file. For every kind of data, 20 files are chosen. It is too complex if they are directly used as the inputs of DNN since the data dimensionality is 20,480, so the data are cut into segments to form the samples. The sampling rate is 20 kHz and the rotation speed is 2000 RPM, so it can be computed that the rotation period is 600 data points per revolution. The size of the segmentation is set to be a quarter of the rotation period, which is 150 data points. Therefore, each file is separated into 136 segments. Then the total number of samples is 10,880 with 150 data points in each segmentation. That is to say, there are 2720 samples for every kind of data. Figure 4 shows an example of one rotation period of normal data and fault data. As we can see, the four kinds of data show in similar tendency and it is hard to classify them just by intuition, therefore, some mathematical method(s) should be implemented for the recognition. A selected dataset description is shown in Table 1.

#### 3.1.3. Training

There are similar tendencies and features in the same kinds of data, which facilitates the classification of data. DNN has the capacity of learning the characteristics and similarities from raw time domain signals. The raw data collected by sensors are taken as inputs of the model and the outputs are data classifications. To select the best neural networks structure, DNN models with different structures are established. An upper bound of neurons is set.

Before the training process, the dataset is divided into three parts: training dataset, testing dataset and validation dataset. The validation dataset is used to select the best trained neural network and to prevent overfitting. The testing dataset is used to calculate the classification accuracy of the networks.

By training these supervised DNN models for hundreds of epochs, model parameters including weights and biases are adjusted to an expected value. In the meantime, the error of this model is decreased to the minimal value and the performance, which is measured by cross entropy, also reaches the expected level. Actually, cross entropy is directly related to errors between model outputs and targets. The cross entropy becomes less when model errors are decreased. The aim of training is to minimize the cross entropy. After training, the five layers model can get an accuracy of 94.4% on the test data when the temporal coherence is not taken into consideration. The simulation models are based on MATLAB. The CPU is an Intel(R) Core(TM) i7-4720HQ @ 2.60 GHZ (Intel Corporation, Santa Clare, CA, USA) and the computation time of the five layers model is 131.161 s for the whole training and testing process. Experimental results show that it can recognize the normal and fault data well, so the model attained good generalization ability. The classification accuracies of these models on the validation dataset (Val) and testing dataset (Test) are shown in Table 2. The confusion matrix of the best model on the testing dataset is shown in Table 3.

The accuracies of the four classifications are measured separately. Outer race faults (label 3) are the easiest to recognize, so their classification accuracy is always 100% no matter how the structure of the model changes. Normal data (label 1) are also easy to recognize, so their accuracies are close 100% too. Inner race fault data (label 2) and roller defect data (label 4) are not so easy to classify, but the accuracies are improved when appropriate models are chosen. Without considering temporal coherence, the four classification accuracies of the five layers DNNs on the testing dataset are 98.9%, 91.4%, 100%, 87.7% and the total accuracy is 94.9%.

### 3.2. Case Western Reserve University (CWRU) Bearing Dataset

#### 3.2.1. Experimental Apparatus and Data Collection

**Figure 5:**
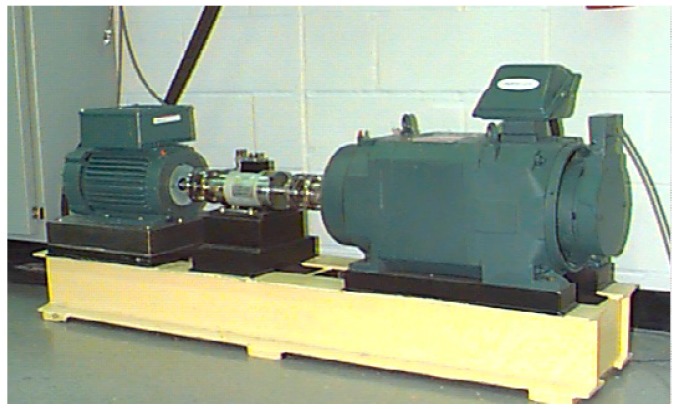
Apparatus for the bearing vibration signal collection of the CWRU bearing dataset.

The experiment apparatus and procedures are shown in Figure 5. The dataset is provided by the Bearing Data Center of Case Western Reserve University [21]. There was a 2 hp motor on the left of the test stand, a torque transducer/encoder in the center and a dynamometer on the right. The control electronics was not shown in the figure. The motor shaft was supported by the bearings in the experiment. Single point faults produced by electro-discharge machining were caused in the test bearings. SKF (Svenska Kullagerfabriken AB, Gothenburgh, Sweden) bearings were used in experiments of 7 mils (1 mil equals to 0.001 inches) diameter bearing faults. The motor rotation speed is 1797 RPM. Accelerometers were attached to the housing with magnetic bases and vibration data were collected using these accelerometers. The accelerometers were placed at the 12 o’clock position at the drive end of the motor housing and vibration signals were collected by a 16 channel DAT recorder. Digital data was collected with the sample rate of 12 kHz for normal vibration and fault vibration. Five kinds of fault vibration signals, including inner race fault, ball fault and three kinds of outer race fault were collected in this experiment. Outer race faults were stationary faults. Placement of the fault with respect to the load zone of bearing had a direct impact on the vibration response of the system. The drive end bearing experiments were conducted with outer race faults located at 3 o’clock which was directly in the load zone, at 6 o’clock which was orthogonal to the load zone, and at 12 o’clock, respectively.

#### 3.2.2. Data Segmentation

**Figure 6:**
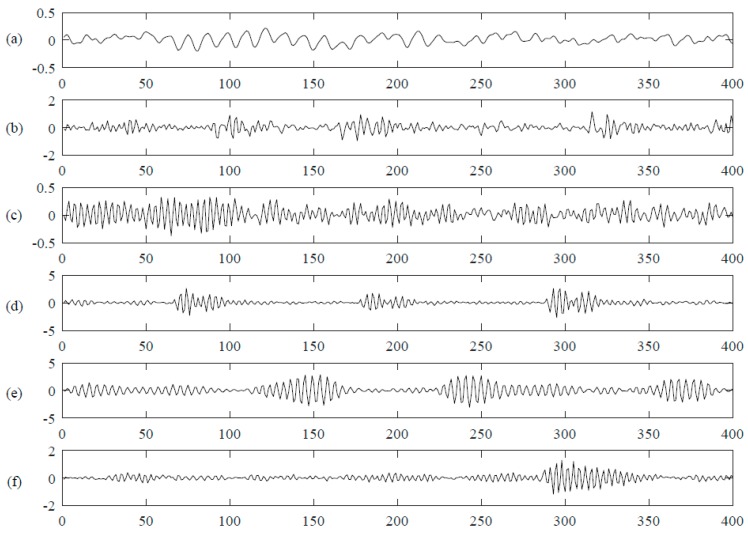
Normal and fault vibration signals are shown in the figure. The x axis is the time series and y axis is the data which is collected by the accelerators on drive end. The first row (**a**) is a part of normal data; (**b**–**f**) are a part of data of inner race fault, ball defect, outer race fault at center @ 6:00, outer race fault at orthogonal @ 3:00, outer race fault @ oppositely @ 12:00, respectively.

For every kind of data, there are at least 121,200 sample points which means that the length of the time series data is at least 121,200. If we directly use them as inputs of deep neural networks, it will be too large to train. The sample rate is 12 kHz and the approximate motor speed is 1797 RPM. Therefore, it can be calculated that there are approximately 401 sample points per revolution. That is to say, the sampling period is approximately 401 points. In this paper, all the data are segmented with the size of one quarter of the sampling period so that the local characteristics can be learnt. There are 1210 samples for every kind of data. Therefore, the total number of samples of the dataset is 7260. The dimension for each sample is 100 and the corresponding target is a six dimensional vector. Examples of six kinds of vibration data are shown in Figure 6 and a selected dataset description is shown in Table 4.

#### 3.2.3. Training

To choose the best neural network parameters, DNN models of different structures are adopted to be trained. In the same way as the method used in the IMS bearing dataset, an upper bound of neurons is set and the dataset is divided into three parts: training dataset, testing dataset and validation dataset. In the DNN model, the first layer is the input layer of which the size is the length of segmentation. The dimension of last layer of DNN is the number of data types. Classification accuracies without considering temporal coherence on test data are shown in Table 5 and the confusion matrix of the best model on the testing dataset is shown as Table 6.

The simulation models are based on MATLAB. The CPU is an Intel(R) Core(TM) i7-4720HQ @ 2.60 GHZ and the computation time of the four layers model is 198.925 s for the whole training and testing process. Experimental results show that normal data (label 1) and outer race fault at orthogonal @ 3:00 (label 5) are easy to recognize, so their classification accuracy are always close to 100%, no matter how many layers are chosen in the DNN. Although the accuracies on other labels are not so great, they can reach around 90%. The four layers model performs best for the whole dataset. The DNN classification accuracies of the six labels are 100%, 88.4%, 90.9%, 95.0%, 99.2% and 92.6%, respectively. The total accuracy is 94.4%.

### 3.3. Performance of the Diagnosis Model Considering Temporal Coherence

In the IMS bearing dataset, the time series data were divided into segments of which the length is a quarter of the sample points in a rotation period. The sampling frequency is 20 kHz so the time length for the segmentation is 7.5 ms. In the CWRU dataset, the inputs of DNN are segments of 100 continuous data points, which is also a quarter of the sample points in a rotation period. They were sampled with the frequency of 12 kHz, so the time length which the model considers is approximately 8.33 ms for every sample. The specifications of these two experiments are shown in Table 7.

In the above two experiments, a quarter of the sample points in a rotation period is used as one training sample. Therefore, the DNN only learns the local characteristics from the time series data and the recognition is drawn out only from local information. As the experiments show, the best total accuracy of DNN on IMS and CWRU bearing data is 94.4% and 94.4%, respectively. The classification accuracy is not so perfect. However, if the outputs of former samples which is computed by the DNN are taken into consideration, the classification accuracy on test data will show a significant progress. The sigmoid function is used as the activation function so the output of DNN is during 0 and 1 and they can be considered as the probabilistic distribution on different kinds of data. The weighted sum of former outputs and current outputs on specific kind of data can be considered as the probability. The outputs of former segmentations are stored. When a new segmentation of sample points occurs, the current DNN output is computed and the recognition is drawn considering former outputs. The recognition can be drawn out when every segmentation of sample points is collected. Although the memory is increased, the time delay remains unchanged.

As shown in Table 2 and Table 5, the total accuracy on the IMS bearing dataset can reach the best value by setting the number of hidden layers of the DNN as five and four, respectively. The best DNN model is adopted to learn the local characteristics of the time series data of above two datasets.

**Figure 7:**
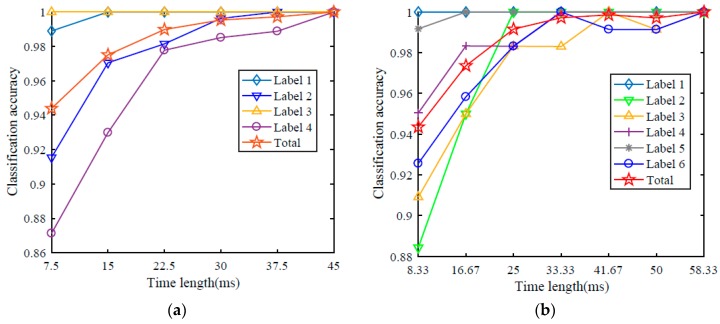
Classification accuracies of IMS (**a**) and CWRU (**b**) bearing dataset considering different time length. The horizontal axis is the time length of data which the model takes into consideration and the vertical axis is the accuracy.

The total accuracy on the IMS bearing dataset is 94.4% when the model only takes 7.5 ms of data into account. 7.5 ms is the time length of one segment of data which is used in the IMS experiment. When one former segmentation output is taken into consideration, the total accuracy jumps to over 97% as shown in Figure 7a. The classification accuracy of a single kind of data also improves when the time length is set to be longer. The total classification accuracy on the IMS bearing dataset can be increased to 100% if the time length is set to be 45 ms, that is to say, five former segments are taken into account.

In the same way, the accuracy on the CWRU bearing dataset also improves a lot with the increase of time length, as shown in Figure 7b. The time length for a single segmentation is 8.33 ms. However, the outer race faults at 12 o’clock (label 6) are not easy to recognize. The classification accuracy of outer race faults at 12 o’clock data can reach over 98% when the time length is set to be longer than 25 ms and it fluctuates with the increase of the time length. The classification accuracies of six kinds of data can all get 100% with enough long time length. When the time length is equal to 58.33 ms, the total classification accuracy on test data is 100%. Figure 7a and Table 8 show the testing dataset accuracies of proposed model considering temporal coherence of IMS bearing dataset. Figure 7b and Table 9 show the accuracies of CWRU bearing dataset.

## 4. Discussion

### 4.1. The Selection of the DNN Structure

The number of neurons in the first layer, i.e., the input layer, is same as the number of data points of one segmentation. The reason is that raw data points collected by the sensors are directly used as DNN inputs. Their dimensionalities must be same. In the IMS bearing dataset, one segment contains 150 data points which is a quarter of the data points collected in one rotation. The sampling rate is 20 kHz and the rotation speed is 2000 RPM so it can be calculated that 600 data points are collected in one rotation. In the CWRU bearing dataset, one segment contains 100 data points which is also approximately a quarter of the data points collected in one rotation. It also can be calculated by the sampling rate 12 kHz and rotation speed 1797 RPM that approximately 401 data points are in one rotation.

The number of neurons in the last layer, i.e., the output layer, is the same as the number of data categories, which includes one normal type and several fault types. In the IMS bearing dataset, there are four types of data, including normal data, inner race fault data, outer race fault data and roller defect data, so the number of output neurons is four. In the CWRU bearing dataset, there are six kinds of data, including normal data, inner race fault data, ball defect data, outer race fault at center @ 6:00 data, outer race fault at orthogonal @ 3:00 data, outer race fault @ oppositely @ 12:00 data, therefore, the number of output neurons is set to six.

There is no strict criterion for selecting the number of neurons in hidden layers. An upper bound of neurons is set. The classification accuracies will be different when these parameters are set to be different. The number of first hidden layers can be set to be larger or smaller than or equal to the number of input layers. The number of neurons in a former hidden layer is simply set to be larger than the number of neurons in the next layer so as to learn more abstract representations. In this paper, the specific numbers of hidden layers are chosen according to the experimental requirements such as the number of first layer and computational complexity.

The number of hidden layers is set to various values and they will give different performance. Comparisons are made for models with different structure. Then the best DNN model is chosen. The experimental results show that the performance of DNN does not always increase with more layers. For example, in the CWRU bearing dataset experiment, the performance of the seven layers model decreased compared with shallower models. Therefore, the DNN structure is selected according to the performance.

### 4.2. Comparision with Other Methods

Table 10 shows classification accuracies of different methods, including Genetic Algorithm with Random Forest [22], Chi Square Features with different classifiers [23], Continuous Wavelet Transform with SVM, Discrete Wavelet Transform with ANN [24], Statistical Locally Linear Embedding with SVM [8] and the method proposed in this paper.

Genetic Algorithm with Random Forest can get an accuracy of 97.81%. Conventional fault diagnosis methods usually select specific features as the classification basis. Some methods can get perfect 100% classification accuracy with the best features. Therefore the features which are extracted from the raw data are the crucial points of these methods. Selecting appropriate features makes a great contribution to the discrimination of the data collected by sensors. For example, chi square feature ranking with SVM can achieve a classification accuracy of 100% when eight features are chosen as the basis. However, the classification accuracy will decrease to 92% when seven features are used. Another example is that the classification accuracy of Discrete Wavelet Transform (Morlet mother wavelet) with ANN can be 96.67%, but it will decrease to 93.33% when the mother wavelet is changed to Daubechies10. Another drawback of these methods is that they do not consider the temporal coherence, i.e., the former data is not taken into account in the current classification process. This paper proposed a DNN-based model without a feature selection process. It learns characteristics directly from raw sensor data and also considers the temporal coherence. The classification accuracy can reach 100%.

## 5. Conclusions

In this paper, a DNN-based model to classify faults is proposed. The raw time series data collected by sensors are directly used as inputs of the proposed model because DNNs are component of learning characteristics from raw sensor data. Conventional fault diagnosis usually focuses on the feature extraction with signal processing methods such as time domain and frequency domain feature representation, EMD, IMF, HHT and DWT. Feature extraction from the time series data is the key point of these approaches, therefore, different data require different feature extraction methods.

The proposed model can autonomously learn the features that are helpful to machinery fault diagnosis. Expertise in feature selection and signal processing is not required. Also, the temporal coherence is taken into consideration. This is a great advantage for prognostics using the proposed method when comparing with conventional fault diagnosis approaches which require of signal processing expertise and extract specific time domain or frequency domain features.

## Figures and Tables

**Table 1:** Description of selected IMS dataset.

Data Type	Number of Samples	Label
Normal	2720	1
Inner race fault	2720	2
Outer race fault	2720	3
Roller defect	2720	4

**Table 2:** Classification accuracies of models with different layers.

Label	3 Layers Model		4 Layers Model		5 Layers Model		6 Layers Model	
	Val	Test	Val	Test	Val	Test	Val	Test
1	98.9%	98.2%	99.3%	98.2%	98.2%	98.9%	97.8%	98.9%
2	92.3%	92.6%	91.9%	91.5%	93.8%	91.5%	90.1%	90.8%
3	100%	100%	100%	100%	100%	100%	100%	100%
4	93.0%	84.9%	90.8%	85.7%	91.9%	87.1%	87.9%	83.5%
Total	96.0%	93.9%	95.5%	93.8%	96.0%	94.4%	93.9%	93.3%

**Table 3:** Confusion matrix of best model (five layers model) on testing dataset.

Actual Classes	Predicted Classes			
	1	2	3	4
1	269	5	0	1
2	0	249	0	34
3	1	0	272	0
4	2	18	0	237

**Table 4:** Description of selected CWRU dataset.

Data Type	Fault Diameter (Inches)	Number of Samples	Label
Normal	0	1210	1
Inner race	0.007	1210	2
Ball	0.007	1210	3
Outer race fault at center @ 6:00	0.007	1210	4
Outer race fault at orthogonal @ 3:00	0.007	1210	5
Outer race fault at oppositely @ 12:00	0.007	1210	6

**Table 5:** Classification accuracies of models with different layers.

Label	3 Layers Model		4 Layers Model		5 Layers Model		6 Layers Model		7 Layers Model	
	Val	Test	Val	Test	Val	Test	Val	Test	Val	Test
1	97.5%	100%	100%	100%	100%	100%	99.2%	100%	98.3%	100%
2	94.2%	91.7%	92.6%	88.4%	98.3%	92.6%	98.3%	95.0%	95.0%	91.7%
3	92.6%	87.6%	93.4%	90.9%	93.4%	83.5%	92.6%	85.1%	92.6%	86.0%
4	97.5%	95.0%	96.7%	95.0%	95.0%	94.2%	93.4%	96.7%	92.6%	94.2%
5	95.9%	98.3%	100%	99.2%	99.2%	98.3%	100%	100%	99.2%	100%
6	91.7%	84.3%	90.9%	92.6%	91.7%	83.5%	90.9%	82.6%	81.0%	71.9%
Total	94.9%	92.8%	95.6%	94.4%	96.3%	92.0%	95.7%	93.3%	93.1%	90.6%

**Table 6:** Confusion matrix of best model (four layers model) on testing dataset.

Actual Classes	Predicted Classes					
	1	2	3	4	5	6
1	121	0	0	0	0	0
2	0	107	0	1	1	2
3	0	2	110	0	0	7
4	0	3	0	115	0	0
5	0	0	0	0	120	0
6	0	9	11	5	0	112

**Table 7:** Specifications of experiments.

Specifications	IMS	CWRU
sampling frequency (kHz)	20	12
rotation speed (RPM)	1797	2000
rotation period (points per round)	600	401
segmentation points	150	100
segmentation on time length (ms)	7.5	8.33

**Table 8:** Classification accuracies considering temporal coherence on IMS dataset.

Time (ms)	Accuracies on Different Labels				
	1	2	3	4	Total
7.5	98.9%	91.5%	100%	87.1%	94.4%
15	100%	97.1%	100%	93.0%	97.5%
22.5	100%	98.2%	100%	97.8%	99.0%
30	100%	99.6%	100%	98.5%	99.5%
37.5	100%	100%	100%	98.9%	99.7%
45	100%	100%	100%	100%	100%

**Table 9:** Classification accuracies considering temporal coherence on CRWU dataset.

Time (ms)	Accuracies on Different Labels						
	1	2	3	4	5	6	Total
8.33	100%	88.4%	90.9%	95.0%	99.2%	92.6	94.4%
16.67	100%	95.0%	95.0%	98.3%	100%	95.8%	97.4%
25	100%	100%	98.3%	98.3%	100%	98.3%	99.2%
33.33	100%	100%	98.3%	100%	100%	100%	99.7%
41.67	100%	100%	100%	100%	100%	99.2%	99.9%
50	100%	100%	99.1%	100%	100%	99.1%	99.7%
58.33	100%	100%	100%	100%	100%	100%	100%

**Table 10:** Classification accuracy of different methods.

Methods	Accuracies
Genetic Algorithm + Random Forest	97.81%
8 Chi Square Features + Random Forest	93.33%
8 Chi Square Features + SVM	100%
7 Chi Square Features + SVM	92%
8 Chi Square Features + Multilayer Perceptron	97.33%
Continuous Wavelet Transform + SVM	100%
Discrete Wavelet Transform (mother wavelet: morlet) + ANN	96.67%
Discrete Wavelet Transform (mother wavelet: daubechies10) + ANN	93.33%
Statistical Locally Linear Embedding + SVM	94.07%
DNN considering temporal coherence	100%
